# Biosynthesis of Se-methyl-seleno-l-cysteine in *Basidiomycetes* fungus *Lentinula edodes* (Berk.) Pegler

**DOI:** 10.1186/s40064-016-2498-5

**Published:** 2016-06-16

**Authors:** M. Klimaszewska, S. Górska, M. Dawidowski, P. Podsadni, J. Turło

**Affiliations:** Department of Drug Technology and Pharmaceutical Biotechnology, Medical University of Warsaw, 1 Banacha Str., 02-097 Warsaw, Poland

**Keywords:** *Lentinula edodes*, Selenium, Se-methyl-seleno-l-cysteine, Selenomethionine

## Abstract

**Background:**

The aim of the current study was to investigate whether the *Basidiomycetes* fungus *Lentinula edodes* can biosynthesize Se-methyl-seleno-l-cysteine, a seleno-amino acid with strong anticancer activity, and to optimize the culture conditions for its biosynthesis. We hypothesize that preparations obtained from Se-methyl-seleno-l-cysteine-enriched mycelia from this medicinal mushroom would possess stronger cancer-preventive properties than current preparations.

**Results:**

By optimizing the concentration of selenium in the culture medium, we increased the mycelial concentration of Se-methyl-seleno-l-cysteine from essentially non-detectable levels to 120 µg/g dry weight. Significantly elevated levels of this amino acid also correlated with significant (twofold) inhibition of mycelial growth. Increases in the concentration of mycelial Se-methyl-seleno-l-cysteine appeared to be highly correlated with the enhanced biosynthesis of selenomethionine and total selenium content in mycelium.

**Conclusions:**

We have demonstrated that in *L. edodes*, enhanced biosynthesis of this non-protein amino acid eliminates excess selenium.

## Background

Nearly four decades ago, a significant inverse correlation between human cancer mortality and dietary selenium intake was reported by Schrauzer and White (Schrauzer and White 1978); the level of selenium in whole blood is directly proportional to dietary selenium intake. Selenium is, therefore, a trace element that appears to function as a key nutrient in cancer chemoprevention (Vinceti et al. 2014). This important health effect is connected to stimulation of the immune system, protection of cells against the effects of free radicals by selenium-dependent antioxidant enzymes, and inhibition of tumor cell growth by selenium metabolites (El-Bayoumy 2001; Rayman 2005; Brozmanova et al. 2010). Despite its benefits, selenium has a narrow margin of safety that limits the doses used in chemoprevention (Reid et al. 2004).

**Fig. 1:**
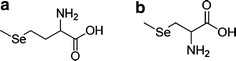
The structures of: **a** l-selenomethionine and **b** Se-methyl**-**seleno-l-cysteine

The bioavailability, tissue distribution, and toxicity of selenium strongly depend on the form ingested. Organic forms of selenium have higher bioavailability and lower toxicity than inorganic species (Brozmanova et al. 2010). For a long time, l-selenomethionine (SeMet) (Fig. 1) was considered to be the organic selenium compound with the most potent anticancer activity (Schrauzer 2000). That opinion seemed to be confirmed by experiments conducted by Clark et al. (Clark et al. 1996), who determined that supplementation with selenium yeast at a dose corresponding to 200 μg of selenium per day appear to decrease overall human cancer mortality by 50 % versus controls. Selenium yeast is a recognized source of organic food-form selenium produced by fermenting *Saccharomyces cerevisiae* (baker’s or brewer’s yeast) in a selenium-enriched media. Since approximately 85 % of selenium in selenium yeast occurs in the form of selenomethionine (Ip et al. 2000), the anticancer effect seemed to be due to selenomethionine (Redman et al. 1998). To confirm these initial findings, the largest-ever prostate cancer prevention trial, the Selenium and Vitamin E Cancer Prevention Trial (SELECT), was designed and carried out. Selenium was administered orally in the form of selenomethionine at a dose of 200 μg for a planned follow-up of a minimum of 7 years and a maximum of 12 years. Initially, SELECT uncovered no reduction in the risk of prostate cancer following ingestion of selenium or vitamin E supplements but did detect a statistically nonsignificant increase in prostate cancer risk with vitamin E ingestion (Lippman et al. 2009). Updated data from SELECT confirmed these preliminary results (Klein et al. 2011; Kristal et al. 2014).

The substantial differences between the results of Clark et al. (Clark et al. 1996) and the results of the SELECT project indicate that selenomethionine was most likely not the molecule primarily responsible for the extremely favorable protective activity of selenium yeast against cancer (Sharma and Amin 2013). The more recent anticancer research focused on Se-methyl**-**seleno-l-cysteine (MeSeCys) (Fig. 1). Numerous studies have demonstrated the efficacy of this non-protein selenoamino acid in preventing and treating cancer (Bhattacharya 2011; Rayman 2005; Rayman 2012). Se-methyl-seleno-l-cysteine in garlic and broccoli has been shown to more efficiently reduce the incidence of mammary and colon cancer in rats than selenomethionine in yeast or broccoli supplemented with selenite (Finley et al. 2001). This effect may be due to substantial differences in the mechanisms of chemoprevention by Se-methyl-seleno-l-cysteine and other selenocompounds (Suzuki et al. 2010).

*Lentinulaedodes* (Berk.) Pegler, the shiitake mushroom, is both medicinal and edible. The antitumor activity of lentinan and other pharmacologically active polysaccharides and polysaccharide-protein complexes in the shiitake extracts results mainly from activation of the host immune system (Zhang et al. 2005). The mechanism by which selenium exerts anticancer and immunomodulatory activities differs from the action of *L. edodes* polysaccharide fractions, but similar pharmacological effects suggest that these agents may act synergistically. Therefore, we hypothesized that high concentrations of the organic forms of selenium in *L. edodes* biomass would increase the immune system-enhancing and anticancer activities of *L. edodes* extracts.

We previously demonstrated that submerged cultivation of *L. edodes* effectively caused selenium from the cultivation medium to accumulate in mycelia in the form of organic compounds such as selenomethionine, selenocysteine and Se-containing polysaccharides (Turło et al. 2007, 2010). The current study sought to determine whether *L. edodes* mycelia grown in medium supplemented with selenium accumulate this element in the form of Se-methyl**-**seleno-l-cysteine and to identify the optimum concentration of selenium in the culture medium for Se-methyl-seleno-l-cysteine biosynthesis.

## Methods

### Microorganisms and cultivation media

The *Lentinula edodes* (Berk.) Pegler strain used in this study was American Type Culture Collection (ATCC, Manassas, USA) strain 48085. The seed culture was grown under the conditions described previously (Suzuki et al. 2010; Zhang et al. 2005).

### Optimization of culture medium for Se-methyl-seleno-l-cysteine biosynthesis

We used the culture medium optimized in our previous research for biosynthesis of SeMet, containing 5 % glucose, 1 % yeast extract, 1.5 % soybean extract, and 0.1 % (w*/*v) KH_2_PO_4_ (Turło et al. 2010). The medium was either not fortified or enriched with selenium to a concentration of 5, 10, 15, 20, or 30 μg*/*mL via the addition of sodium selenite. Fermentation medium was inoculated with the seed culture at 10 % (v*/*v) and cultivated at 26 °C on a rotary shaker at 110 rev/min for 14 days. Mycelia were harvested via filtration, freeze dried, and used to determine the concentration of Se-methyl-seleno-l-cysteine.

### Extraction of Se-methyl-seleno-l-cysteine

Against decomposition of Se-methyl-seleno-l-cysteine, extraction was performed at 4 °C (Arnault and Auger 2006). Twenty milliliters of 50 mM HCl were added to 0.5 g of homogenized mycelia and sonicated for 20 min. The mixture was then stirred for 6 h at 4 °C and stored at 4 °C. The samples were filtered under reduced pressure, the filtrate was freeze-dried, and the resulting dry extract was stored at 4 °C in the dark. Dried extract was redissolved in water to the appropriate concentration for determination of MeSeCys.

### Analytical determination of Se-methyl-seleno-l-cysteine

Determination of Se-methyl-seleno-l-cysteine was performed with high-performance liquid chromatography (HPLC) of *o*-phthaldialdehyde derivative, compared with the retention time of a Se-methyl-seleno-l-cysteine standard (Sigma, Saint Louis, USA). Our method was a modification of reported previously (Turło et al. 2007).

Samples were prepared by dissolving 0.1 g of mycelium extract in 10 ml of HPLC-grade water. The assay was performed using a calibration curve (external standard). Se-methyl-seleno-l-cysteine recovery under the conditions of extraction and analysis was determined by subjecting the standard to the same procedure as the samples. HPLC was carried out using a Shimadzu Manufacturing INC (Canby, USA) gradient system (SCL-10AVP controller, two LC-10ATVP pumps, CTO-10ACVP oven) with a fluorescence detector (LaChrom L-7480, Merck-Hitachi, Darmstadt, Germany). C_18_ column 250 × 4 mm; Phenomenex (Torrance, USA), Luna 2 with appropriate guard column was used. Eluent A consisted of 50 mM sodium acetate buffer (pH 7.0) containing 0.1 % acetonitrile while eluent B was methanol. The gradient breakpoints (min/%B) were (0/40), (6/45), (12/45), (20/63), (38/63), and (49/100). The temperature was 32 °C, the injection volume was 20 μL, and the flow rate was 1.2 mL/min. The excitation wavelength was 340 nm, and the emission wavelength was 435 nm. Se-methyl-seleno-l-cysteine retention occurred at 14.9 min.

### Analytical determination of selenomethionine

Selenomethionine content was determined by reverse-phase HPLC of *o*-phthaldialdehyde derivative (OPA method) as previously described (Turło et al. 2007). The conditions of the analysis were as follows:

Eluent A was 100 mM sodium acetate buffer pH 7.2 containing 0.1 % of acetonitrile, eluent B was methanol.

The gradient breakpoints were as follows (min/%B): (0/45), (6/45), (12/50), (20/63), (38/63), (49/100), (55/100). Temperature was 32 °C, injection volume 20 μL and flow rate 1.2 mL/min. The chosen excitation wavelength was 340 nm and emission wavelength 435 nm. The selenomethionine retention time was 40.2 min.

### Analytical determination of total selenium

Reverse-phase HPLC of mineralized samples was used to determine the total selenium content, as previously described (Turło et al. 2009). Namely, a 0.1 % solution of diaminonaphthalene (DAN) in 0.1 M HCl was prepared and stored in the dark at 4 °C. The pH of each of digested sample dissolved in 8 mL of Suprapur water was adjusted to 1.8–2 by adding HCl or 7 M NH_3_·H_2_O, as needed. One milliliter of DAN was added to each sample and the mixtures were heated for 45 min at 75 °C. After cooling, 3 mL of cyclohexane was added and the samples were shaken vigorously for 1 min to extract the fluorescent piazselenol. Prior to RP HPLC determination the samples were stored in the dark. The sample solutions were diluted with cyclohexane as needed. A blank digest was manipulated similarly for use as a control. Selenium standard solutions (6.25–1000 ng Se/mL) were prepared under the same conditions. The fluorescence was a linear function of the concentration of Se in the tested range (correlation coefficient, R = 0.999).

The RP-HPLC conditions were as follows: eluent, acetonitrile: flow rate, 1.4 mL/min; temperature, 25 °C; injection volume, 20 μL. For fluorimetric analysis, the excitation wavelength was 378 nm and emission wavelength was 557 nm. The piazselenol retention time was 3.1 min.

### Statistical analysis

Data were analyzed using one-way analysis of variance or *t* Student test with the statistical package STATISTICA 10 (StatSoft). Arcsine transformation was used to equal variances before one-way analysis of variance. Post-hoc analysis was performed with the least-significant difference test. Cell viability data were analyzed using *t* Student test. Differences were considered to be significant when *p* value <0.05.

## Results

### Extraction and determination of Se-methyl-seleno-l-cysteine

Cold extraction (with 50 mM HCl at 4 °C) was chosen in order to avoid decomposition of Se-methyl-seleno-l-cysteine in the resulting extract. Se-methyl-seleno-l-cysteine is stable at room temperature, but extraction at high temperature cause its substantial loss. In general, organic selenium species remain stable for up to 1 year in aqueous solution at pH 4.5 when stored at 4–20 °C in the dark (Amoako et al. 2007). Extraction overnight at 4 °C in 50 mM HCl (2:1 v/w) was previously used for routine quantification of Se-methyl-seleno-l-cysteine in plant samples (Ellis et al. 2004). The current applications are the first use of this cold method for the isolation of this non-protein amino acid from fungal biomass. When developing the extraction method, we have tested the recovery of MeSeCys under different conditions. Under low-temperature (4 °C) extraction conditions the recovery of MeSeCys was 98.8 % (±2.3 %), while at a temperature of 100 °C (Soxhlet extraction)—only 4 % (±1.2 %), n = 5.

**Fig. 2:**
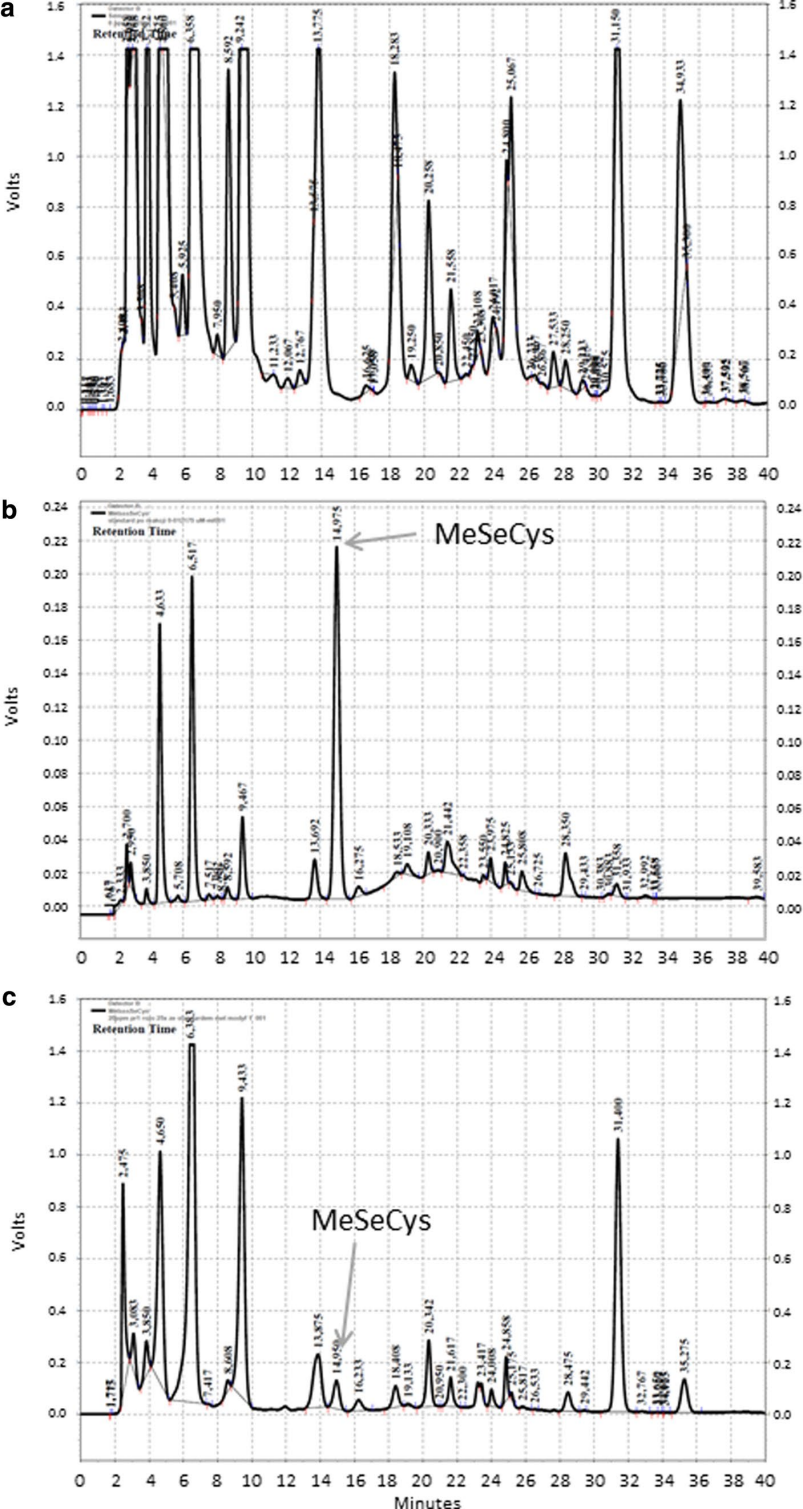
Representative chromatograms of: **a** extract from not Se-enriched mycelium, **b** mixture of extract from not Se-enriched mycelium with MeSeCys standard solution, **c** extract from mycelium cultivated in medium enriched with 20 μg*/*mL Se

Determination of amino acids with high-performance liquid chromatography (HPLC) of *o*-phthaldialdehyde derivatives is described as a method of good selectivity and efficiency (Hammel et al. 1997; Bartolomeo and Maisano 2006). The standard HPLC equipment and relatively cheap C_18_ columns used are significant advantages of this approach. The detection limit for most of the OPA amino acid derivatives is in range 25–50 fmol (Hammel et al. 1997). In our previous research, we successfully applied this method for determination of selenomethionine in mycelial hydrolysates (Turło et al. 2007). Currently, this approach was successfully used for determination of Se-methyl-seleno-l-cysteine. The detection limit (LOD) for the OPA Se-methyl-seleno-l-cysteine derivative was 63 fmol, limit of quantification (LOQ)—211 fmol. The fluorescence was a linear function of the concentration of MeSeCys in the tested range of 0–25 nmol/mL (correlation coefficient R = 0.999, equation for standard curve was y = 2.182E−06x, where y-MeSeCys concentration, x—peak area). The reproducibility of the method was assessed by calculating the relative standard deviations for MeSeCys standard solutions (n = 5); the values ranged from 1.35 to 4.1 %. The standard deviation of the blank was used to estimate the detection and quantification limits of the complete procedure. The lowest determined MeSeCys concentration in the mycelial extract was 79 pM, thus it was nearly 400-fold higher than limit of quantification (LOQ). The Fig. 2 shows the representative chromatograms of the extract from not Se-enriched mycelium, the extract from mycelium cultivated in medium enriched with 20 μg*/*mL Se and the mixture of extract from not Se-enriched mycelium with MeSeCys standard solution.

### Effect of medium supplementation on mycelial growth

The medium that we selected on the ground of the previous research was associated with a satisfactory yield of mushroom mycelium (10 g of dry weight/1 L of the medium after 10 days of cultivation) (Table 1). According to our previous results increasing the selenium concentration (in the form of sodium selenite) had a dose-dependent inhibitory effect on mycelial growth (Turło et al. 2007, 2010). When the concentration selenium in the medium was greater than 15 μg*/*mL, the mycelial growth decreased.

**Table 1:** Relationship between the selenium concentration in the culture medium and the total selenium, selenomethionine and Se-methylselenocysteine contents in *L. edodes* mycelia

Se concentration in medium (μg*/*mL)	Concentration in mycelium (μg/g)			Mycelium growth (g/L)
	Se	SeMet	MeSeCys	
0	0.6_a_ (0.1)	uql_a_	uql_a_	10.4_a_ (0.6)
5	169.2_b_ (3.1)	61.4_b_ (2.0)	7.2_b_ (2.0)	9.8_a_ (0.7)
10	345.55_c_ (24.9)	117.0_c_ (25.9)	28.1_c_ (0.6)	10.5_a_ (0.9)
15	592.3_d_ (29.6)	275.0_d_ (56.3)	67.9_d_ (1.9)	11.5_b_ (0.7)
20	1080.5_e_ (52.6)	672.3_e_ (88.1)	120.5_e_ (16.7)	6.6_c_ (0.7)
30	1973.6_f_ (78.6)	424.9_f_ (49.1)	108_e_ (6.9)	6.3_c_ (0.6)

Standard error are given in parentheses. n = 5 replicates

Within columns, values bearing different subscript letters were significantly different from each other (p < 0.05)

Concentration values for mycelium components are expressed in μg/g of mycelial dry weight

Mycelium growth is given in grams of mycelial dry weight/1 L of culture medium

### Biosynthesis of Se-methyl-seleno-l-cysteine in *L. edodes*

We wanted to evaluate whether the presence of sodium selenite in medium stimulated biosynthesis of MeSeCys by mycelium of *L. edodes* and, if so, to find the optimal concentration. The concentration of Se-methyl-seleno-l-cysteine rose from not detectable content in mycelium cultivated in non-enriched medium to 120 μg/g dry mass of mycelium grown in medium enriched with 20 μg*/*mL of Se. When the concentration of Se in the medium was greater than 20 μg*/*mL, Se-methyl-seleno-l-cysteine content in the mycelium decreased (Table 1).

### Biosynthesis of selenomethionine in L. edodes

The amount of selenomethionine in the harvested mycelial biomass increased with the increasing concentration of selenium. The optimum concentration was 20 μg*/*mL, which provided a concentration of 672 μg/g dry mass in the harvested mycelium. Higher concentrations of selenium inhibited selenomethionine biosynthesis (Table 1).

## Discussion

Despite the sizeable interest, the metabolism of selenium by higher fungi remains poorly understood. This process is most widely characterized in plants, motivating us to compare our data on the biosynthesis of Se-methyl-seleno-l-cysteine in *L. edodes* with reports concerning plants. According to Ellis and Salt (Ellis and Salt 2003), plants primarily take up selenium as selenate or selenite, which are then metabolized via the sulfur assimilation pathway to yield selenomethionine, selenocysteine, and other selenium analogs of various sulfur metabolites. Non-specific incorporation of selenoamino acids into proteins is thought to contribute to selenium toxicity (Ellis et al. 2004). A proposed mechanism of selenium tolerance in plants is the specific conversion of potentially toxic selenoamino acids into non-protein derivatives such as Se-methyl-seleno-l-cysteine (Wang et al. 1999). Our previous studies indicated that *L. edodes* can incorporate selenium into the proteins, mainly in the form of selenomethionine (Turło et al. 2007). Toxicity occurred when selenomethionine was present at >300 µg/g of fungal biomass and a total selenium content of 1100 µg/g, resulting in substantial inhibition of mycelial growth (Turło et al. 2010). We also found that excess selenium is eliminated via its reduction to elemental selenium (Turło et al. 2010), what was 2 years later confirmed by Tsivileva et al. (2012).

**Fig. 3:**
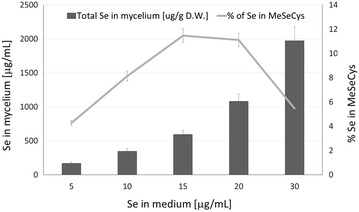
Percentage of selenium accumulated in the form of Se-methyl-seleno-l-cysteine against the total content of selenium in the mycelium

In the current study by optimizing the concentration of selenium in the culture medium, we increased the mycelial concentration of Se-methyl-seleno-l-cysteine from essentially non-detectable levels to 120 μg/g D.W. (Table 1). However, only 4–11 % of the selenium accumulated in the form of Se-methyl-seleno-l-cysteine (Fig. 3).

**Fig. 4:**
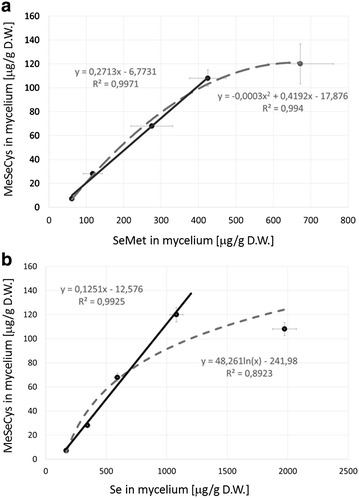
Correlation of Se-methyl-seleno-l-cysteine content in mycelium with **a** selenomethionine. content and **b** total selenium content in cultivated mycelia

Increases in the concentration of mycelial Se-methyl-seleno-l-cysteine appeared to be highly correlated with the enhanced biosynthesis of selenomethionine. MetSeCys content in cultivated mycelia also rose as total selenium content increased (Table 1). As it was stated above, the MeSeCys contents in mycelium well correlate with the total selenium and SeMet. The presented graph (Fig. 4) indicates for selenium content up to 1000 μg/g and the MetSeCys up to 300 μg/g the correlation is linear (r = 0.992, p < 0.005, r = 0.997, p < 0.001) while for the higher concentrations nonlinear.

It suggests that elimination of excess selenium via SeMet and MetSeCys biosynthesis is not the sole way of detoxification in the case of high selenium concentration. As mentioned above, in high selenium concentrations, the excess selenium is also eliminated via its reduction to elemental red selenium (Turło et al. 2010; Tsivileva et al. 2012). It can be assumed that, as in plants, incorporation of selenomethionine into the structures of mushroom proteins likely contributes to selenium toxicity: mycelial growth was significantly reduced (p < 0.0005) when the concentration of selenomethionine in the mycelia was >300 µg/g of dry mass. This effect was accompanied by a nearly twofold increase in the concentration of Se-methyl-seleno-l-cysteine (Table 1). These observations are consistent with the assumption that the biosynthesis of non-protein derivatives of selenium is a method of detoxification.

## Conclusions

Here we have demonstrated that in the *Basidiomycetes* fungus *L. edodes*, enhanced biosynthesis of the non-protein amino acid Se-methyl-seleno-l-cysteine eliminates excess selenium. Significantly elevated levels of this amino acid correlated with significant (twofold) inhibition of mycelial growth. By optimizing the concentration of selenium in the culture medium, we increased the mycelial concentration of Se-methyl-seleno-l-cysteine from essentially non-detectable levels to 120 µg/g dry weight. The further problem is to examine whether, according to our hypothesis, high concentration of MeSeCys in extracts of the fungus increases the anti-cancer effect.

## Abbreviations

MeSeCys: Se-methyl-seleno-l-cysteine, Se-methyl selenocysteine (CID:105024)
SeMet: l-selenomethionine or 2-amino-4-methylselanyl-butanoic acid (CID:147004)
RP-HPLC: reverse-phase high-performance liquid chromatography
LOD: limit of detection
LOQ: limit of quantification
D.W.: dry weight
Uql: under quantification limit
